# Changes in psychological distress during conflict escalation in an adult population-based cohort in the Gaza Strip (2020–2025): a longitudinal analysis

**DOI:** 10.1016/j.eclinm.2025.103647

**Published:** 2025-11-24

**Authors:** Curdin Brugger, Bassam Abu Hamad, Jan Hattendorf, Mirko S. Winkler, Nicole Probst-Hensch

**Affiliations:** a Swiss Tropical and Public Health Institute, Kreuzstrasse 2, 4123, Allschwil, Switzerland; b University of Basel, Petersplatz 1, 4001, Basel, Switzerland; c Al-Quds University, Gaza City, Palestine

**Keywords:** War, Mental health, Psychological distress, Cohort, Gaza

## Abstract

**Background:**

The Gaza Strip has experienced prolonged conflict and blockade, straining infrastructure, basic services, and daily life. These chronic stressors pose a severe threat to mental health. The escalation to war in October 2023 led to widespread displacement, destruction, and loss of life. While cross-sectional studies before the war suggest high psychological distress, longitudinal data documenting the temporal course of mental health during conflict escalation are lacking.

**Methods:**

This study uses longitudinal data from 677 adults (aged at least 40 years) who participated in three self-report household surveys (2020, 2023, 2025) in the Gaza Strip. The three surveys were carried out in the governorates of Gaza, North Gaza, and Rafah, and responses were gathered from March 18 to July 15, 2020; January 24 to March 7, 2023; and January 12–28, 2025, respectively. The primary endpoint, mental health status, was assessed in all three survey rounds using the 12-item General Health Questionnaire (GHQ-12). Items were scored dichotomously (0/1), resulting in a total GHQ-12 score ranging from 0 to 12, with higher scores indicating greater psychological distress. We applied a conservative threshold of GHQ-12 score >6 for defining high psychological distress, consistent with previous studies in the Gaza Strip. Descriptive analyses documented temporal changes in GHQ-12 scores and items. A mixed-effects logistic regression model estimated the association between survey year and high psychological distress, adjusting for socio-demographic variables.

**Findings:**

A total of 677 participants took part in all three surveys (from 2980 in 2020 and 1547 in 2023). In 2020, 49%, 31%, and 20% of the 677 retained participants lived in Gaza, North Gaza, and Rafah governorates, respectively. 51% (n = 347) of the participants were women, and the majority (70%) were aged 40–59 years. High psychological distress (GHQ-12 score >6) increased from 19.5% in 2020 and 17.4% in 2023 to 67.2% in 2025. Adjusted models showed 12 times higher odds of psychological distress in 2025 compared with 2020 (OR = 12.45; 95% CI: 9.01–17.20). This increase did not differ across socio-demographic subgroups, although older adults and those with secondary or higher education were less likely to exhibit high psychological distress.

**Interpretation:**

While our study cannot establish causality, the recent tripling of severe psychological distress evolved from a background of an exceedingly high mental health burden before October 2023. Our findings underscore the relevance of providing long-term psychosocial and mental health services, including strengthening resilience, to prevent long-term consequences for the current and next generations in Gaza and other conflict-affected populations. Future research should examine the long-term mental health trajectories and resilience, including intergenerational impacts.

**Funding:**

10.13039/501100002992UK’s Department for International Development, 10.13039/501100000265Medical Research Council, 10.13039/501100000269Economic and Social Research Council, 10.13039/100010269Wellcome Trust, Gaza Resilience Program of the International Committee of the Red Cross, and Department of Epidemiology and Public Health, Swiss TPH.

**Research in context**

**Evidence before this study**

Existing research on the prevalence of mental health disorders and psychological distress in the Gaza Strip is mostly limited to cross-sectional surveys conducted before the escalation to war in October 2023. We searched PubMed for English publications between Oct 7, 2023, and July 28, 2025, using the search terms (“Gaza” [All Fields]) AND (“mental health” [All Fields]) AND (2023/10/07:2025/07/28 [Date–Publication]). This search identified five cross-sectional studies conducted since the start of the current war. One study included comparative cross-sectional data obtained in 2022 and again during the war for a sample of medical students but did not assess the same individuals longitudinally. All identified studies point to very high levels of mental burden in the Gaza Strip during the current war. The study using 2022 pre-war data for comparison found a significant increase in all measured mental health outcomes. However, no study to date has captured within-person changes in mental health from before to during the current war. No longitudinal or cohort studies of the general population with pre-war baseline data have been identified in the Gaza Strip, and such data remains limited in war-affected populations globally.

**Added value of this study**

This study employs a longitudinal design to follow the mental health of adults in the Gaza Strip across three time points, including baseline data collected before the outbreak of the current war in October 2023, up to January 2025. To our knowledge, it is the first population-based study to track individual-level changes in psychological distress in Gaza over multiple years. Our findings demonstrate a threefold increase in the prevalence of severe distress by 2025 compared to 2020. This temporal perspective adds critical new insight by building on pre-war data to quantify the mental health impact of war, a study design rarely available in Gaza or other war-affected regions.

**Implications of all the available evidence**

Whilst acknowledging this study design cannot establish causality, our combined body of evidence across three surveys shows high psychological distress in the Gaza Strip even before the current war, followed by a sharp rise to an extremely high mental health burden since October 2023. This highlights the urgent need to expand mental health and psychosocial support services, alongside broader efforts to strengthen individual and community resilience in the Gaza Strip and other war-affected regions. The severe and widespread nature of the distress underscores the importance of sustained, inclusive, and context-sensitive mental health interventions that extend well beyond the end of active conflict. Further studies are needed to track long-term mental health outcomes and the development of resilience over time, including potential intergenerational effects, in populations affected by conflict.

## Introduction

Exposure to armed conflict is strongly associated with adverse mental health outcomes. A 2019 meta-analysis estimates that 22% of individuals living in post-conflict settings in low-income and middle-income countries experience mental health conditions such as depression, anxiety disorders, post-traumatic stress disorders (PTSD), bipolar disorder, or schizophrenia, with nearly 10% suffering from moderate to severe mental disorders.^1^ Exposure to violence and displacement, along with war-related chronic stressors (such as economic instability, poverty, disrupted social systems and networks, unreliable basic services, unemployment, and lack of future prospects) are all strong risk factors for poor mental health.^2^, ^3^, ^4^, ^5^ In prolonged conflicts, the mental health burden can increase significantly and affect not only individuals but entire populations and possibly future generations.^6^^,^^7^

The Gaza Strip has experienced decades of conflict with varying intensity, and a longstanding blockade severely restricting movement and access to essential goods.^8^ Repeated escalations have led to cycles of destruction and reconstruction of basic infrastructure, as well as thousands of deaths and injuries.^8^ In 2017, nearly 50% of Gazans were unemployed.^9^ By 2023, before the current escalation to war in October 2023, 80% of households had only intermittent electricity with an average of 10 h of daily electricity, and nearly 20% of households were water insecure.^2^^,^^9^^,^^10^ The escalation since October 2023 has intensified these challenges with extensive destruction of infrastructure, homes, and essential services.^11^ By January 2025, 90% of Gazans were internally displaced, 47% of the hospitals were only partially functional, 92% of housing was destroyed or damaged, and more than 45,000 Palestinians were killed, with some studies suggesting underreporting by 41%.^11^^,^^12^ Cross-sectional studies conducted in the Gaza Strip during the current war have reported mental disorder prevalence (such as for anxiety, depression, and PTSD) ranging from 55% to 99% across different population groups.^3^^,^^13^, ^14^, ^15^, ^16^, ^17^ From 2022 to 2024, extremely severe depression and anxiety symptoms in medical students in the Gaza Strip increased from 12.4% to 61.4% and 28.5% to 44.3%, respectively.^16^

The General Health Questionnaire (GHQ-12) is a validated screening tool widely used in epidemiological studies, including in conflict and humanitarian settings,^13^^,^^18^, ^19^, ^20^, ^21^ to assess general psychological distress and identify individuals at risk for common mental disorders.^22^^,^^23^ Although the tool does not provide a clinical diagnosis, it serves as a validated indicator of probable mental health problems at the population level. Different scoring approaches and threshold cut-offs are used to determine risk levels depending on the GHQ-12 version, study context, and population characteristics.^22^^,^^23^ For January to September 2023, UNRWA (United Nations Relief and Works Agency for Palestine Refugees in the Near East) reported that 27% of screened patients in the Gaza Strip had a high GHQ-12 score, indicating a need for further care.^18^ A survey conducted in mid-2024 among adolescents found that 43% had a high GHQ-12 score.^13^

Conducting studies during active conflict is generally challenging, and longitudinally following up on study participants adds even more challenges.^24^ Thus, although previous studies examined mental health in the Gaza Strip either before or during the current conflict, to our knowledge, this is the first longitudinal cohort study in Gaza with pre-war baseline mental health data, following the same individuals across both periods. To fill this gap, the current study utilises three rounds of household survey data collected from the same individuals in 2020, 2023, and 2025; thus assessing psychological distress before, shortly before, and during the most recent conflict escalation. The primary objectives of this study are to describe the temporal course of psychological distress, analyse changes over time using the GHQ-12, and to provide evidence to support mental health service planning, mitigating potential long-term and intergenerational effects.

## Methods

### Study design and ethics

This study is based on longitudinal data nested in repeated cross-sectional household surveys conducted in the Gaza Strip. The three surveys were carried out in the governorates Gaza, North Gaza, and Rafah from March 18 to July 15, 2020; January 24 to March 7, 2023; and January 12 to 28, 2025. The first survey, conducted in 2020, focused on non-communicable diseases and therefore only included participants above 40 years. The 2023 and 2025 surveys follow-up with the same cohort of participants, and therefore, this study focuses on participants aged 40 years and older. The survey methods in 2020^21^ and 2023^25^ have been described elsewhere. We followed the STROBE guidelines to ensure comprehensive reporting of our study.

Ethical approval for the 2020 survey was granted by: the Imperial College Research Ethics Committee (20IC5733), the American University of Beirut Institutional Review Board, the Gaza Helsinki Committee (PHRC/HC/483/19); for the 2023 survey by: the Gaza Helsinki Committee (PHRC/HC/1141/22), the Ethikkommission Nordwest und Zentralschweiz (AO_2022-00076), the International Committee of the Red Cross internal Ethics Review Board (LDPCORE 23/00006-CGB/bap); for the 2025 survey by: the Gaza Helsinki Committee (PHRC/HC/483/19). Informed consent was obtained from all participants before data collection (2020, 2023: written; 2025: oral).

### Participants

The initial survey in 2020 used a sampling approach designed to produce a representative sample of the Gaza Strip population across all five governorates. In each governorate, clusters were randomly selected, and within each cluster, 15 households were randomly chosen for the survey. In 2023, a stratified random sampling method was applied to select a sub-sample from the original participants in the governorates Gaza, North Gaza, and Rafah. In 2025, participants were eligible if they were 40 years or older, had participated in at least one of the previous surveys, had consented to future contact and provided a valid phone number. Households were randomly selected from the 905 households participating in the 2023 survey. The 2025 sample aimed to maintain proportionality by study area (North Gaza, Gaza, and Rafah) and gender. For each area, a randomised calling list was generated. Households were contacted in this order until the target sample size of 700 participants (ideally 350 women and 350 men) was reached. A total of 777 individuals were sampled to participate in 2025. For 73 of these, the survey was not completed. Among the 704 participants who completed the survey, 677 had participated in all three surveys (Appendix p 2). In the 2020 and 2023 surveys, more than one participant per household could be included. In 2025, only one eligible adult per household was sampled, maintaining the household-based sampling frame while focusing on individual-level participants.

### Procedures

Trained interviewers involved in the 2020 and 2023 in-person surveys also conducted the 2025 survey. The structured telephone interviews lasted approximately 30–45 min per participant. Data collection was conducted using SurveyCTO (https://www.surveycto.com), a secure electronic data collection platform. Established and validated questionnaires were used where possible. Where applicable, identical survey items were used across all three rounds. Instruments with validated Arabic versions were used as is. Newly developed or adapted items were translated into Palestinian Arabic, with back-translation into English performed when needed to ensure accuracy.

The primary endpoint, mental health status, was assessed in all three survey rounds using GHQ-12 (Table 1). Items were scored dichotomously (0/1), resulting in a total GHQ-12 score ranging from 0 to 12, with higher scores indicating greater psychological distress. We applied a conservative threshold of GHQ-12 > 6 for defining high psychological distress, consistent with prior studies in the Gaza Strip by UNRWA and other research, reflecting a high probability of clinically relevant distress.^13^^,^^18^ For descriptive purposes and selected figures, we additionally applied the less conservative threshold of GHQ-12 > 2, often used in general population studies.^22^^,^^23^ While no formal cultural validation was conducted in the present survey, prior research in Palestine and other Arabic-speaking regions suggests that the GHQ items are well-understood and culturally appropriate for capturing psychological distress.^13^^,^^18^^,^^26^^,^^27^

**Table 1:** The 12 questions of the General Health Questionnaire (GHQ-12) and the item abbreviations used in figures and throughout the manuscript.

Item	Abbreviation	GHQ-12 English
1	Able to concentrate	Been able to concentrate on what you're doing?
2	Lost sleep	Lost much sleep over worry?
3	Play a useful part	Felt you were playing a useful part in things?
4	Make decisions	Felt capable of making decisions about things?
5	Under strain	Felt constantly under strain?
6	Overcome difficulties	Felt you couldn't overcome your difficulties?
7	Enjoy activities	Been able to enjoy your normal day-to-day activities?
8	Face problems	Been able to face up to your problems?
9	Depressed	Been feeling unhappy and depressed?
10	Losing confidence	Been losing confidence in yourself?
11	Felt worthless	Been thinking of yourself as a worthless person?
12	Felt happy	Been feeling reasonably happy, all things considered?

Each question was introduced with “In the past two weeks, have you …” and answered with “Yes” or “No.” The Arabic translation of the GHQ-12 is provided in the Appendix (p 3).

The main predictor of interest, survey year (2020, 2023, 2025), was added as a variable to each data set during data analysis. The covariates at baseline, namely socio-demographic characteristics, included gender (male/female), age, civil status, refugee status, recent work, and education, and were self-reported. The governorate of the household before the current war was recorded by the interviewer. Age (<60/≥60 years), civil status (married/single), refugee status (refugee/non-refugee), and education (none or basic/primary/secondary or higher) were grouped for statistical analysis. Detailed variable descriptions and group definitions are shown in the Appendix (p 4).

### Statistical analysis

No formal sample size calculation was performed. The 2025 target (n = 700) was based on financial and logistical feasibility, and proportional allocation by gender and governorate. Analyses were conducted using R (version 4.5.1) and RStudio (version 2025.05.1).

To assess potential selection bias, socio-demographic characteristics and baseline GHQ-12 scores (2020) were compared between participants included in the main longitudinal analysis (n = 677) and those not participating in all three surveys (n = 2303).

Missing data were handled using available-case analysis without imputation. No sampling weights or attrition adjustments were applied, as the primary aim was to assess within-responder change over time rather than population-level prevalence.

For descriptive comparisons of temporal change, the single GHQ-12 items, the GHQ-12 score percentiles, and the grouped GHQ-12 scores (0–2; 3–6; 7–12) were compared across the three surveys. Figures and tables in the main text include the 677 participants who participated in all three surveys. Additional figures and tables in the Appendix present GHQ data for different sample subsets, including the full cross-sectional samples (2020, n = 2980; 2023, n = 1615; 2025, n = 704), retained and not-retained participants, and subgroup analyses by gender.

Regression analyses were restricted to the 677 retained participants to model within-individual change over time. To assess the association of year of assessment and socio-demographic factors with psychological distress (GHQ-12 > 6) modelled as a binary variable, we fitted a mixed-effects logistic regression model (R-package: lme4). Results are presented as odds ratios (ORs) with 95% confidence intervals (CIs). Unadjusted models were first run for each variable separately to estimate crude associations. We then fitted adjusted models that included survey year, governorate, gender, age group, civil status, refugee status, recent work, and education as fixed effects. Interaction terms between survey year and gender, and survey year and age group, were tested a priori to explore differential trends over time. Other potential interactions were not tested to avoid an increasing risk of chance findings without strong theoretical justification. Random intercepts for individuals and sampling clusters accounted for within-responder and within-cluster correlation. Time-varying covariates such as displacement, loss of housing, or unemployment were not included because these were not consistently measured across all survey waves. Our primary focus was on population-level temporal trends, rather than the analysis of specific time-varying exposures.

As a post-hoc sensitivity analysis, we re-ran the main mixed-effects logistic regression model using inverse probability weighting (IPW) to assess the robustness of findings to potential attrition bias. The probability of completing all three survey waves was estimated using baseline variables (gender, age group, governorate, and refugee status), and stabilised inverse probability weights were applied in the model.

Changes in single GHQ-12 items between the three surveys, the percentage of negative answers at each survey, as well as the change between surveys, were calculated and graphically presented.

### Role of the funding source

The funders of the 2020 and 2023 surveys had no leading role in study design, data collection, data analysis, data interpretation, or report writing, whereas the funder of the 2025 survey (Department of Epidemiology and Public Health, Swiss TPH) had a leading role in all these aspects.

## Results

Of the 677 individuals who responded in all three surveys, before the current war, 49%, 31%, and 20% lived in Gaza, North Gaza, and Rafah governorates, respectively. 51% (n = 347) of the participants were women, more than two-thirds (70%) were aged 40–59 years, and 95% were married. Thirteen percent had no finished formal education, 49% had completed elementary and preparatory education, and 38% had completed secondary or higher education (Table 2). Socio-demographic characteristics and categorised GHQ-12 score distribution were not materially different between participants retained in all three surveys (n = 677) and those not retained (n = 2303). Retained participants were slightly younger (aged 40–59 years: 70% vs. 60%) and included a slightly higher proportion of men (49% vs. 45%). The proportion with high baseline psychological distress (GHQ-12 > 6) was similar between groups (19.5% vs. 23.4%; Appendix p 5–6).

**Table 2:** Socio-demographic characteristics of study participants (based on 2020 data) and the prevalence of high psychological distress (General Health Questionnaire score, GHQ-12 > 6) in 2025 by characteristics (n = 677).

Baseline characteristics from 2020	Overall	High GHQ-12 (>6) in 2025
	n = 677 (100%)	n = 455 (67%)
**Governorate**		
North Gaza	207 (31%)	162 (78%)
Gaza	333 (49%)	185 (56%)
Rafah	137 (20%)	108 (79%)
**Gender**		
Female	347 (51%)	240 (69%)
Male	330 (49%)	215 (65%)
**Age group**		
40–59	476 (70%)	324 (68%)
60–89	201 (30%)	131 (65%)
**Civil status**		
Married	641 (95%)	432 (67%)
Single	36 (5.3%)	23 (64%)
**Refugee status**		
Refugee	476 (70%)	333 (70%)
Non refugee	201 (30%)	122 (61%)
**Worked in the past 30 days**		
Yes	132 (19%)	86 (65%)
No	545 (81%)	369 (68%)
**Education**		
None or basic	86 (13%)	65 (76%)
Primary	331 (49%)	230 (69%)
Secondary and higher	260 (38%)	160 (62%)

To assess the temporal change in the prevalence of psychological distress, we first compared the GHQ-12 score distribution by survey year using the full cross-sectional samples from each wave (Appendix p 7). In 2020 (n = 2980), 22.5% of the participants had a GHQ-12 > 6 and 19.6% had a score <3, similar to 2023 (n = 1615; 19.5% > 6; 21.0% < 3). In 2025 (n = 704), this pattern shifted, with 67.6% of participants scoring >6 and only 1.8% < 3.

**Fig. 1:**
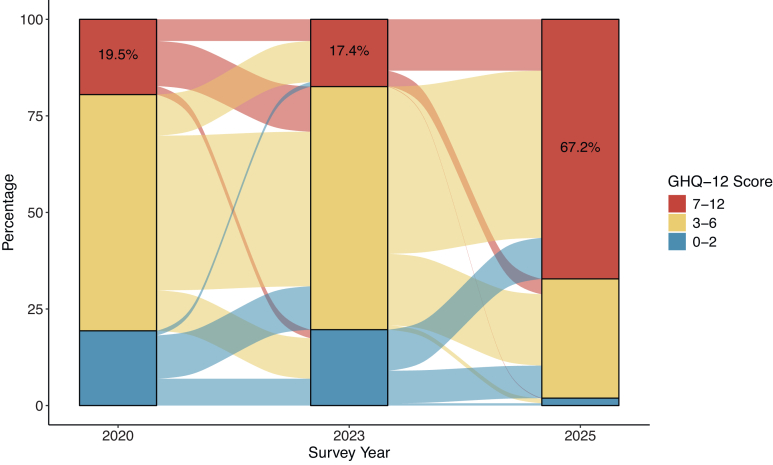
Change of the General Health Questionnaire (GHQ-12) scores from baseline (2020) through follow-ups in 2023 and 2025 among 677 participants from Gaza, North Gaza, and Rafah.

Second, we described the temporal trend of GHQ-12 scores at the individual level, including participants present in all three surveys (n = 677; Fig. 1). Regarding temporal change in GHQ-12 scores between 2020 and 2023, we observed a similar proportion of participants with an increase versus a decrease in GHQ-12 score, leading to relatively similar distributions of GHQ-12 score groups. In contrast, after 2023, the most severe GHQ-12 score group more than tripled from 17.4% to 67.2%, an absolute increase of 49.8 percentage points. The least severe GHQ-12 group was close to non-existent in 2025 (1.9%). The proportion of participants with a decrease in GHQ-12 from 2023 to 2025 was minimal. Gender-separated figures are presented in the Appendix (p 8); patterns of change in GHQ-12 scores were not materially different between men and women. Comparing percentiles of the GHQ-12 score further illustrates these distributional changes (Appendix p 9). The median score remained stable between 2020 and 2023 (median = 6) but increased to 9 in 2025. The 90th percentile increased from 7 in 2023 to 10 in 2025, while the 10th percentile rose from 3 to 5 over the same period.

**Fig. 2:**
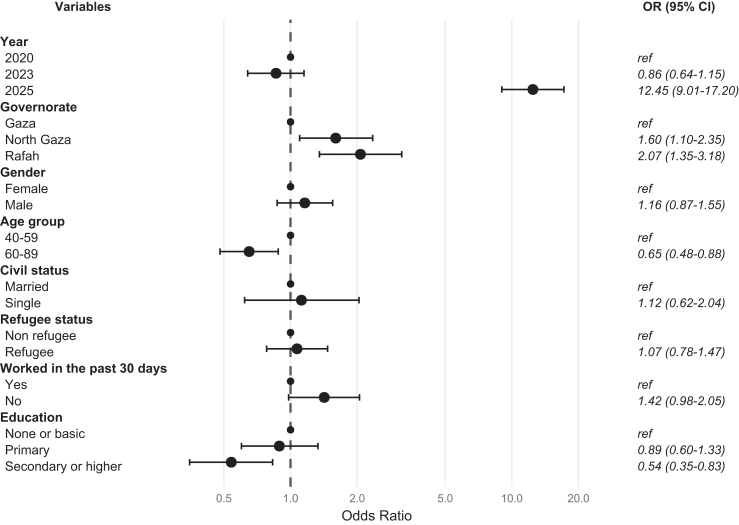
Forest plot showing mutually adjusted odds ratios (ORs) for the association between survey year and baseline socio-demographic characteristics with a high level of psychological distress (General Health Questionnaire, GHQ-12 > 6). Estimates are from a mixed effect logistic regression model accounting for clustering at the individual and cluster levels (n = 677). Circles represent ORs and horizontal lines indicate 95% confidence intervals (CIs). Reference categories are indicated as “ref” in the figure.

Upon regression of psychological distress (GHQ-12 > 6 versus ≤6) on survey year, participants in 2025 (67.2%, 455/677) had 12.44 times higher odds (95% CI: 8.99–17.20) of psychological distress compared to 2020 (19.5%, 132/677; Appendix p 10), with an absolute difference of 47.7 percentage points. After adjusting for socio-demographic factors, this association remained equally strong (OR = 12.45; 95% CI: 9.01–17.20; Fig. 2), corresponding to over twelve times higher odds of high psychological distress in 2025 compared to 2020.

Participants in North Gaza (37.7%, 234/621) had 1.60 times higher odds of psychological distress (95% CI: 1.10–2.35) compared to those in Gaza (30.1%, 301/999, difference of 7.6 percentage points). Similarly, participants in Rafah (41.4%, 170/411, difference of 11.3 percentage points) had significantly higher odds (OR = 2.07; 95% CI: 1.35–3.18). Participants aged 60–89 (30.5%, 184/603) had 35% lower odds of psychological distress (OR = 0.65; 95% CI: 0.48–0.88) compared to those aged 40–59 (36.5%, 521/1428, difference of 6 percentage points). Likewise, participants with secondary or higher education (30.1%, 235/780) had reduced odds (OR = 0.54; 95% CI: 0.35–0.83) compared to those with no or basic education (38.4%, 99/258, difference of 8.3 percentage points).

No significant difference (OR = 1.16; 95% CI: 0.87–1.55) was observed between male (34.4%, 341/990) and female participants (35.0%, 364/1041, difference of 0.6 percentage points). No interactions were found between survey year and either sex or age group, suggesting that the temporal increase in psychological distress occurred similarly across sex and age categories.

Post-hoc sensitivity analysis using IPW produced estimates closely aligned with the primary analysis. The adjusted odds of psychological distress in 2025 compared with 2020 were 12.45 (95% CI 9.01–17.20) in the unweighted model (Figs. 2) and 13.78 (95% CI 9.97–19.06) in the IPW-weighted model (Appendix p 11).

**Fig. 3:**
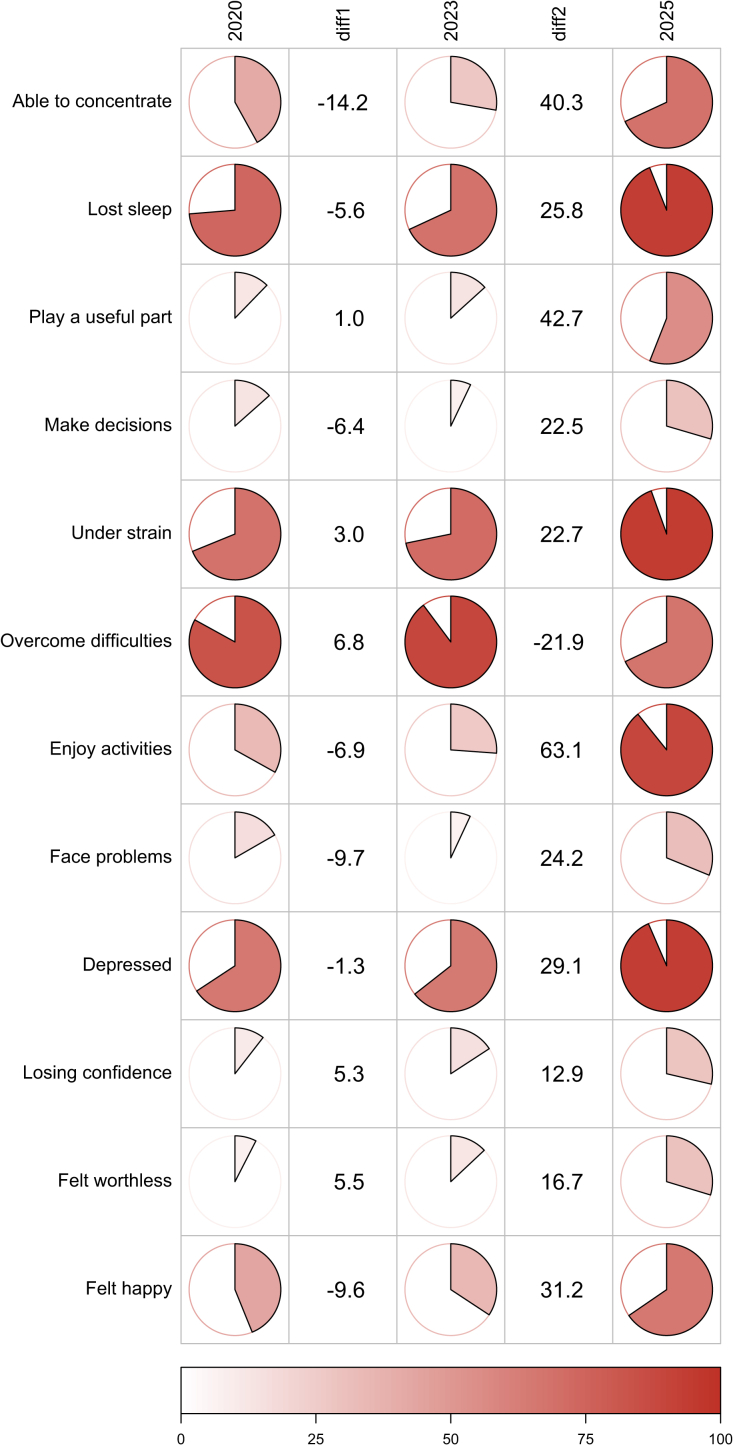
Survey-specific distribution of responses to individual General Health Questionnaire (GHQ-12) items and their change between surveys among 677 participants from Gaza, North Gaza and Rafah in 2020, 2023, and 2025. The GHQ-12 questions are scored as yes or no, with the pie charts showing the percentage of participants providing negative responses. Darker shades of red represent higher percentages of negative responses, indicating greater psychosocial distress. Changes between surveys are presented as diff1 (percentage point change from 2020 to 2023) and diff2 (percentage point change from 2023 to 2025). Larger and more positive diff values signify greater increases in distress-related responses.

We additionally assessed the temporal change in negative answers to the single GHQ-12 items, indicating more psychological stress, among participants retained across all three surveys (n = 677; Fig. 3). Before the war, in 2020 and 2023, the highest percentage of negative answers was observed for the items Lost sleep, Under strain, Overcome difficulties, and Depressed, implying that these items were important contributors to psychological distress before the war. In contrast, the lowest percentage of negative answers was observed for the items Play a useful part, Make decisions, Face problems, Losing confidence, and Felt worthless, suggesting that these items were less influential on psychological distress before the war. The distribution of negative answers to single items substantially changed with the war, as evidenced by the much larger percentage point changes in negative answers between 2023 and 2025 compared to 2020 and 2023. From 2023 to 2025, we observed a major increase ranging from 12.9 to 63.1 percentage points in negative responses in all items except the item Overcome difficulties, for which the percentage of negative answers decreased by 21.9 percentage points. The highest increase (63.1 percentage points) was observed for the item Enjoy activities, indicating a decreasing trend in the percentage of participants still having access to or enjoying activities. The distribution of single-item answers in the full cross-sectional samples of each survey was not materially different (Appendix p 12).

## Discussion

The longitudinal course of mental health in the study participants shows a dramatic deterioration in psychosocial well-being since the escalation to war in October 2023. Already prior to the conflict in 2020 and 2023, nearly one in five participants met the criteria for severe mental health problems, more than what is often observed in post-conflict settings.^1^ By early 2025, 15 months into the ongoing war, this proportion had more than tripled, with nearly 70% of participants scoring above the conservative GHQ-12 threshold applied (>6 score).

This marked shift occurred across all subgroups, irrespective of age or gender, indicating a widespread and indiscriminate mental health burden that coincides with the ongoing war. While our study cannot establish causality, the sharp increase in mental distress between 2023 and 2025 coincides temporally with the outbreak and continuation of the current war. The magnitude of the sharp increase in psychosocial distress from before the war to January 2025, with odds in 2025 12 times higher than in 2020, is consistent with a strong temporal association with conflict escalation. Indeed, the timing of the increase suggests a likely contribution of war-related exposures, including multiple displacements, economic deprivation, significant barriers to accessing health care and widespread loss of essential infrastructures. Notably, over 99% of the participants in our study reported at least one displacement event by early 2025, with the median number of displacements being four. The combination of high rates of displacement occurring in a context of widespread civilian casualties, destruction, lack of protected shelters, food insecurity, limited formal social protection services, and trauma likely contributed to the rapid deterioration of mental health among the study population.^3^^,^^11^, ^12^, ^13^, ^14^, ^15^, ^16^^,^^28^, ^29^, ^30^

Compared with evidence on conflict and post-conflict populations elsewhere, the observed 2025 mental health status of adults in Gaza, which is in agreement with other recent studies showing exceedingly high levels of anxiety, depression and PTSD, appears among the poorest recorded.^1^^,^^3^^,^^13^, ^14^, ^15^, ^16^, ^17^ Cross-sectional evidence on psychological disorders in other populations in acute conflicts points to prevalence rates of 11%–55%.^5^^,^^31^, ^32^, ^33^ One study of the Israeli population following the onset of the war has reported increases in anxiety, depression, and PTSD relative to before, although the prevalence of severe mental health outcomes remains substantially lower than that observed in the Gaza Strip.^33^ Unlike in our study, where psychological distress was widespread across the population, a second study of the Israeli population found uneven impacts, with higher risks observed among individuals with pre-existing mental health conditions, ethnic minorities, and women^32^; possibly reflecting the near-universal exposure to psychological trauma and shocks in Gaza. Longitudinal evidence capturing mental health trajectories from pre-conflict to wartime remains generally scarce, and research is needed to understand these trajectories and to identify protective factors, including culturally rooted concepts such as sumud (steadfastness),^34^^,^^35^ that may buffer against long-term psychological decline.

The implications of widespread mental distress in conflict settings are profound. For adults, sustained psychological distress is linked to increased risk of cardiovascular disease, impaired immune function, and mortality.^7^^,^^20^^,^^21^^,^^31^ The high burden of poor mental health may also undermine health care engagement, economic participation, and social cohesion, which are all key elements of individual and community recovery.^28^^,^^30^ For children, the impact of living with chronically distressed caregivers is well-documented, with increased risks for behavioural problems, emotional dysregulation, and impaired development.^4^^,^^7^^,^^36^^,^^37^ Given that nearly 70% of adults in our study scored above the GHQ-12 threshold in 2025, these intergenerational risks are likely to be substantial in the study population. Noting that intergenerational risks/trauma were not directly investigated herein. This concern is reinforced by a recent study among adolescents in Gaza conducted earlier in the war, which found that 43% exceeded the GHQ-12 threshold. Although somewhat lower than in our adult cohort, this still represents a strikingly high level of distress in young people and suggests that both caregivers and their children are concurrently affected.^13^ The intergenerational consequences could extend to the children of these children, perpetuating cycles of trauma, disadvantage, and vulnerability if not addressed through long-term, multi-sectoral intervention.^6^^,^^7^^,^^37^

Mental health rehabilitation will be crucial for Gaza's recovery, including the health and well-being of future generations.^6^^,^^7^^,^^30^ Delivering mental health services in Gaza faces immense obstacles, including a shortage of trained professionals, ongoing insecurity, and extensive damage to all infrastructure, including health.^7^^,^^30^ Recent work highlights how these barriers require integrated, context-sensitive strategies that combine service delivery with structural and community-based resilience-building.^3^^,^^4^^,^^30^^,^^38^ Future mental health interventions and rehabilitation efforts should build on, strengthen, and collaborate with existing organisations and programmes to enhance reach and effectiveness in Gaza. In this context, our single-item GHQ-12 results suggest that certain domains of coping and resilience, such as maintaining decision-making capacity, facing problems, or preserving confidence, remained relatively intact in a proportion of participants even during the conflict. These findings may point to protective factors at the individual and community level, including social support, adaptive problem-solving, and religious and culturally rooted coping strategies (particularly the Palestinian concept of sumud), that warrant further study to understand subgroup differences in trajectories of distress. Mental health rehabilitation must extend beyond clinical services to include broad-based and inter-sectoral psychosocial support, safe and stable living conditions, religious practices, and inclusive community rebuilding.^1^^,^^4^^,^^28^^,^^29^^,^^38^ Interventions must be inclusive, given that all subgroups exhibited a high burden and equally drastic recent surge of psychological distress. Targeted support for children is critical to prevent intergenerational transmission of trauma.^3^^,^^4^^,^^6^^,^^7^

Key strengths of this study include its rare longitudinal design in a conflict zone, with three assessments capturing both pre-war and during-war periods. The large, population-based sample (stratified by gender and governorate) enhances representativeness, while consistent use of the validated GHQ-12 ensured comparability across waves. The high retention of participants (677 across all surveys) further strengthened the study. Maintaining a cohort in a conflict-affected setting also demonstrates the feasibility of mental health monitoring in crisis contexts and points to the relevance of establishing population-based cohorts in countries before a crisis evolves.

Limitations include the restriction to adults aged 40 years and older and the geographical focus on only three governorates, which limit generalisability, in particular given the comparatively young age of the Gaza population. Attrition may have introduced bias; however, baseline characteristics, including GHQ-12 scores, were similar between retained and not retained participants. In addition, we conducted a sensitivity analysis using IPW to account for potential attrition bias (Appendix p 11). The results were consistent with the primary analysis, with no meaningful changes in the estimates. The observational design limits causal inference, and the lack of detailed, time-varying mediator data constrains analysis of specific pathways. Residual confounding, for example by socio-demographic status or exposure to violence, may persist. It is also notable that, although the GHQ-12 is a widely used screening tool for psychological distress, it is not a clinical diagnostic tool. Which might constrain the interpretability of our findings. Furthermore, responses to the GHQ-12 may be influenced by cultural context, which should be considered when interpreting the results. Reliance on self-reported data and the use of different interview methods (in-person versus phone interviews) can introduce reporting and measurement bias. No imputation was performed for missing values, which were minimal in key variables.

Our findings underscore the value of longitudinal data in following mental health of individuals through different phases of conflict. They highlight the substantial burden of mental health challenges faced by war-affected populations globally, emphasising the critical need to prioritise mental health protection and rehabilitation in these settings. Acknowledging resilience and cultural concepts, such as sumud, in the discussion allows for a nuanced framing that recognises both the challenges faced and the strengths within the population, without implying causality. The evidence underscores the urgency of protecting and rehabilitating mental health of current and future generations affected by severe conflicts globally and in the Gaza Strip. The process needs to be accompanied by research, particularly through mixed-methods approaches to deepen understanding of risks, needs, and resilience factors for implementing efficient interventions. Additionally, future research should explore the long-term trajectories of mental health recovery after the end of active war, especially beyond immediate crisis periods, to include intergenerational impacts.

## Contributors

NPH, MSW, BAH and CB conceptualised and designed this study. BAH and CB were responsible for project administration. CB and NPH wrote the first draft of the manuscript with input from MSW, BAH and JH. CB and JH developed the methodology; CB conducted the statistical analysis. CB and JH accessed and verified the underlying data and the analysis. All authors have access to all the data and share responsibility for the decision to submit for publication.

## Data sharing statement

The data used in the analysis and findings of this study are available from the corresponding author MSW, upon reasonable request.

## Editor note

The Lancet Group takes a neutral position with respect to territorial claims in published maps and institutional affiliations.

## Declaration of interests

We declare no competing interests.

## Appendix A Supplementary data

Supplementary Figures and Tables

Translated Abstract
