# Supplementary material for: Changes in psychological distress during conflict escalation in an adult population-based cohort in the Gaza Strip (2020–2025): a longitudinal analysis
Source: eClinicalMedicine. 2025 Nov 24;90:103647. doi: 10.1016/j.eclinm.2025.103647 (PMC12766424; doi:10.1016/j.eclinm.2025.103647)
Supplement: Supplementary Figures and Tables [file mmc1.pdf]

Supplementary appendix

Supplement to: Changes in psychological distress during conflict escalation in an adult population-based cohort in the Gaza Strip (2020–2025): a longitudinal analysis

Table of contents

Participant flowchart 2
GHQ-12 questionnaire, including translation 3
Variable description 4
Socio-demographic characteristics for 2020 and lost to follow-up 5
Distribution of baseline GHQ-12 score for different groups 6
Distribution of GHQ-12 score in each survey year 7
Longitudinal changes in GHQ-12 scores by gender 8
GHQ-12 Score Percentiles across Survey Years 9
Unadjusted regression models 10
Sensitivity analysis using inverse probability weighting 11
Answers to GHQ-12 items for the full sample in each survey 12

Participant flowchart

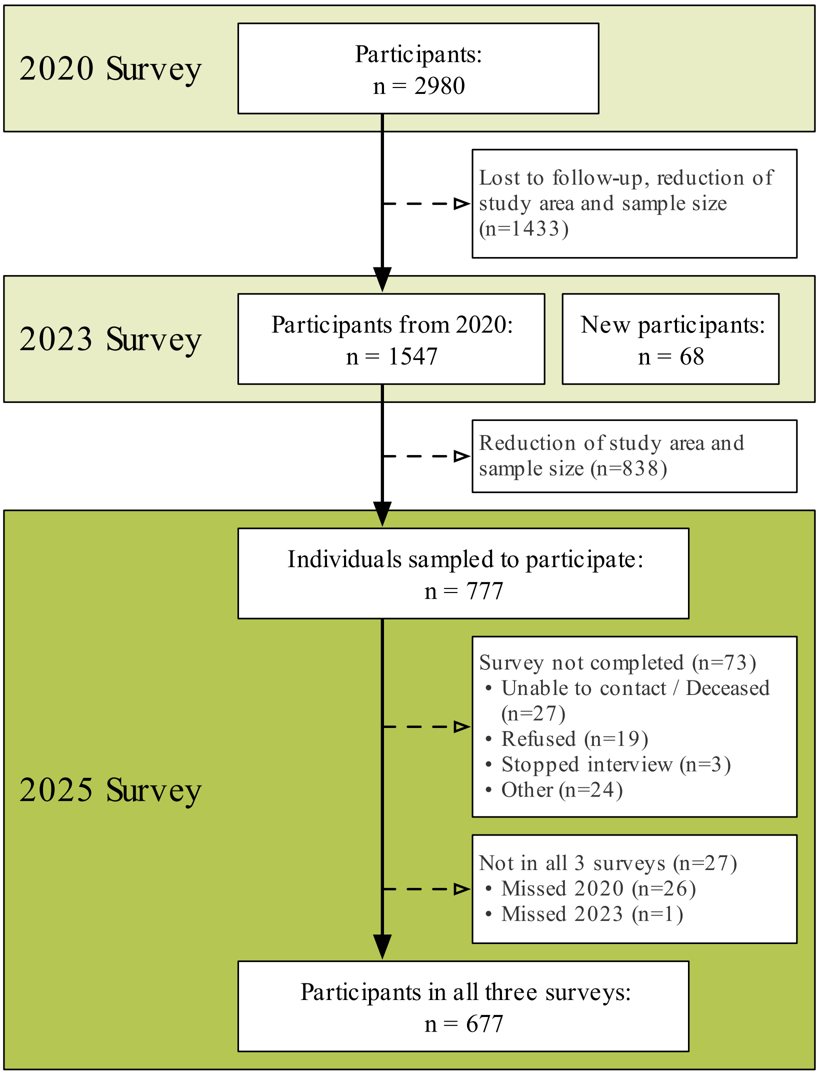

Supplementary Figure 1: Participant flow from the 2020 baseline survey through follow-ups in 2023 and 2025 in Gaza, North Gaza and Rafah governorates.

GHQ-12 questionnaire, including translation

Supplementary Table 1: Original English wording and Arabic translation of the 12 items of the General Health Questionnaire (GHQ-12) and the item abbreviations used in figures and throughout the manuscript. Each question was introduced with "In the past two weeks, have you…" and participants responded with "Yes" or "No".

Item	Abbreviation	GHQ-12 English	GHQ-12 Arabic
1	Able to concentrate	Been able to concentrate on what you're doing?	كنت قادراً على التركيز في كل ما تعمله؟
2	Lost sleep	Lost much sleep over worry?	اضطرب نومك بسبب القلق؟
3	Play a useful part	Felt you were playing a useful part in things?	شعرت بانك قمت بدور فعال في مهامك الحياتية؟
4	Make decisions	Felt capable of making decisions about things?	شعرت أنك قادر على اتخاذ القرارات اتجاه مهامك الحياتية؟
5	Under strain	Felt constantly under strain?	شعرت دوماً أنك متوتر / مضغوط؟
6	Overcome difficulties	Felt you couldn't overcome your difficulties?	شعرت أنك قادر على تجاوز المصاعب؟
7	Enjoy activities	Been able to enjoy your normal day-to-day activities?	كنت قادر عل الاستمتاع بنشاطاتك اليومية المعتادة؟
8	Face problems	Been able to face up to your problems?	كانت لديك القدرة على مواجهة مشاكلك؟
9	Depressed	Been feeling unhappy and depressed?	راودك الشعور أنك غير سعيد ومكتئب؟
10	Losing confidence	Been losing confidence in yourself?	كنت تفقد الثقة في نفسك؟
11	Felt worthless	Been thinking of yourself as a worthless person?	فكرت بأنك شخص عديم الفائدة؟
12	Felt happy	Been feeling reasonably happy, all things considered?	شعرت بسعادة بدرجة معقولة ناتجة عن مواقف تستدعي ذلك؟

Variable description

Supplementary Table 2: Description of variables included in the analyses and rationale for category selection. GHQ-12 = General Health Questionnaire, UNRWA = United Nations Relief and Works Agency for Palestine Refugees in the Near East, IQR = interquartile range.

Variable	Original value or question	Categories used in analysis	Reason for categories
**GHQ-12**	Score range = 0-12	0-2	We used two cut-offs:<br>GHQ-12 >6 is commonly applied in the Gaza Strip by UNRWA and other studies.<br>GHQ-12 >2 is often used in general population studies.
		3-6	
		7-12	
**Survey year**	2020, 2023, 2025	Original value	
**Governorate**	North Gaza, Gaza, Rafah	Original value	
**Gender**	Male / Female / Other	Male / Female	One man and one woman were identified per household; categories used were Male / Female. No participant identified as “Other” and no further probing was undertaken due to cultural sensitivity.
**Age**	Median = 54, IQR = 48 – 61<br>Range = 40 – 83	40-59	Age was categorised into two groups to ensure a sufficient sample size within each category.
		60-89	
**Civil status**	Currently married	Married	We recoded civil status into a binary variable (married / single). This decision was based on distribution, as most non-married categories represented less than 1% of the sample individually, with 'widowed' being the most common at 9%.
	Separated	Single	
	Divorced		
	Widowed		
	Single / Never married		
	Engaged / Marriage contract		
**Refugee**	Registered refugee	Refugee	Registered and unregistered refugees were combined into the 'refugee' category, as unregistered refugees comprised < 1% of the sample. We defined refugees as descendants of Palestinian refugees from the 1948 war.
	Unregistered refugee		
	Not a refugee	Non refugee	
**Recent work**	Did you work during the past 30 days?	Yes / No	
**Education**	Illiterate	None or basic	The education variable reflects the highest level of education completed by participants. Categories were grouped into three levels based on levels used in previous studies and to ensure adequate sample sizes within each group, while also capturing meaningful distinctions in educational attainment relevant to the local context.
	Can read and write		
	Elementary	Primary	
	Preparatory		
	Secondary	Secondary and higher	
	Associate diploma		
	Bachelor's degree		
	Higher diploma		
	Master's degree		
	PhD		

Socio-demographic characteristics for 2020 and lost to follow-up

Supplementary Table 3: Socio-demographic baseline characteristics of 2020 study participants from Gaza, North Gaza and Rafah, comparing the full sample in 2020 with those who did not participate in all three surveys and those who did participate in all three surveys.

Baseline Characteristics 2020	Full baseline sample 2020	Not participating in all 3 surveys	Participating in all 3 surveys
	N = 2980	N = 2303	N = 677
**Governorate**			
North Gaza	827 (28%)	620 (27%)	207 (31%)
Gaza	1576 (53%)	1243 (54%)	333 (49%)
Rafah	577 (19%)	440 (19%)	137 (20%)
**Gender**			
Female	1611 (54%)	1264 (55%)	347 (51%)
Male	1369 (46%)	1039 (45%)	330 (49%)
**Age group**			
40-59	1853 (62%)	1377 (60%)	476 (70%)
60-89	1124 (38%)	923 (40%)	201 (30%)
90+	3 (0·1%)	3 (0·1%)	0 (0%)
**Civil status**			
Married	2677 (90%)	2036 (88%)	641 (95%)
Single	303 (10%)	267 (12%)	36 (5%)
**Refugee status**			
Refugee	1984 (67%)	1508 (65%)	476 (70%)
Non refugee	996 (33%)	795 (35%)	201 (30%)
**Worked in the past 30 days**			
Yes	538 (18%)	406 (18%)	132 (19%)
No	2441 (82%)	1896 (82%)	545 (81%)
Missing	1	1	
**Education**			
None or basic	448 (15%)	362 (16%)	86 (13%)
Primary	1348 (45%)	1017 (44%)	331 (49%)
Secondary and higher	1184 (40%)	924 (40%)	260 (38%)

Distribution of baseline GHQ-12 score for different groups

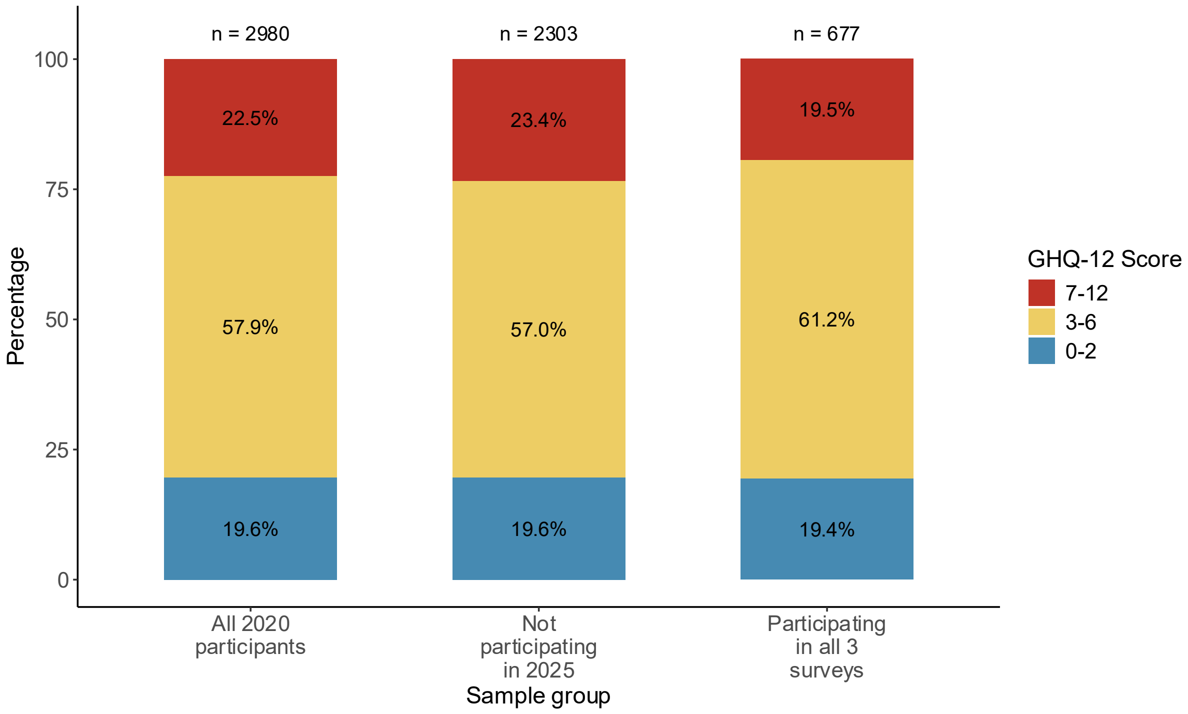

Supplementary Figure 2: Distribution of baseline General Health Questionnaire (GHQ-12) scores in 2020 across different participant groups. Bars show the proportion of participants in each of the three GHQ-12 score categories at baseline, shown for (1) the full sample from Gaza, North Gaza, and Rafah; (2) participants who did not take part in all three surveys; and (3) participants who completed all three surveys.

Distribution of GHQ-12 score in each survey year

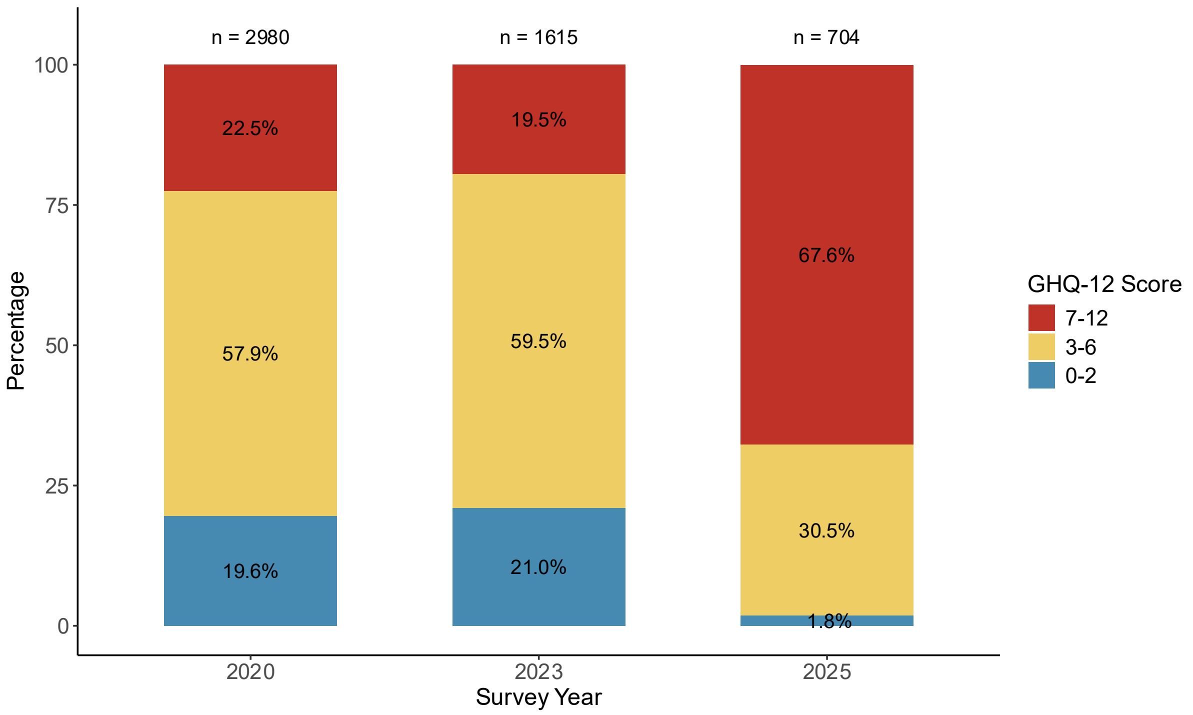

Supplementary Figure 3: Distribution of General Health Questionnaire (GHQ-12) scores by survey year. Bars show the proportion of participants in each of the three GHQ-12 score categories for each survey year, using the full available sample from Gaza, North Gaza, and Rafah.

Longitudinal changes in GHQ-12 scores by gender

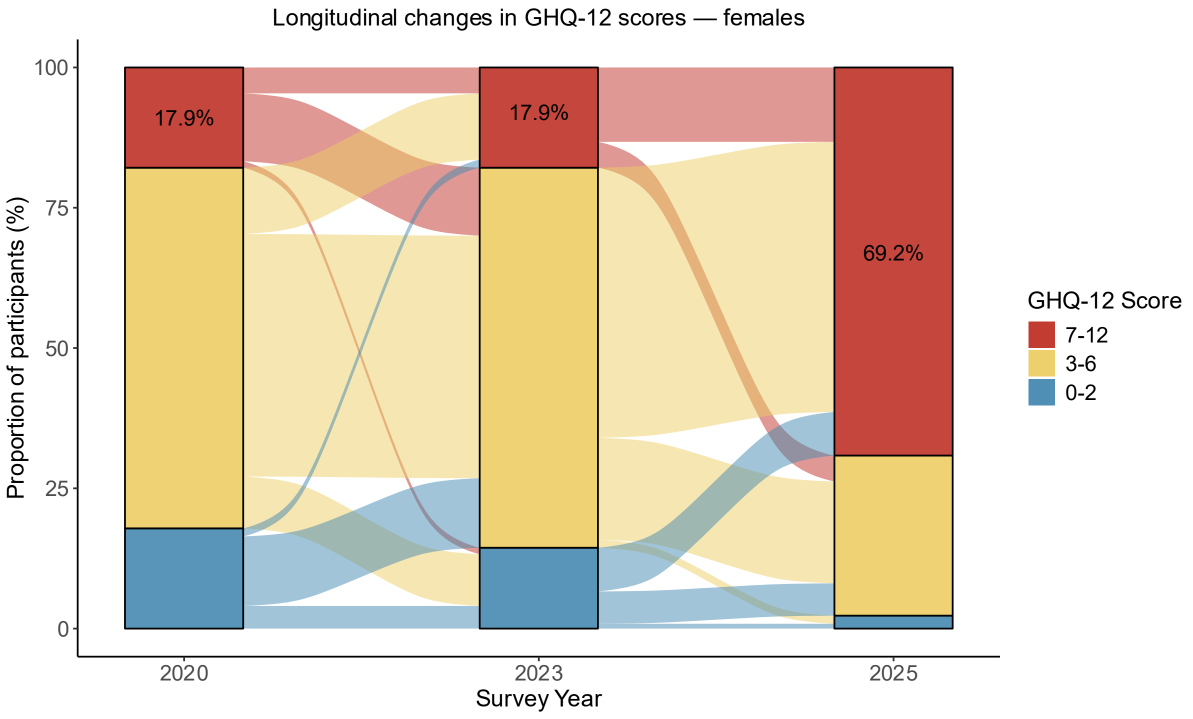

Supplementary Figure 4: Change of General Health Questionnaire (GHQ-12) scores from baseline (2020) through follow-ups in 2023 and 2025 among 347 female participants from Gaza, North Gaza, and Rafah.

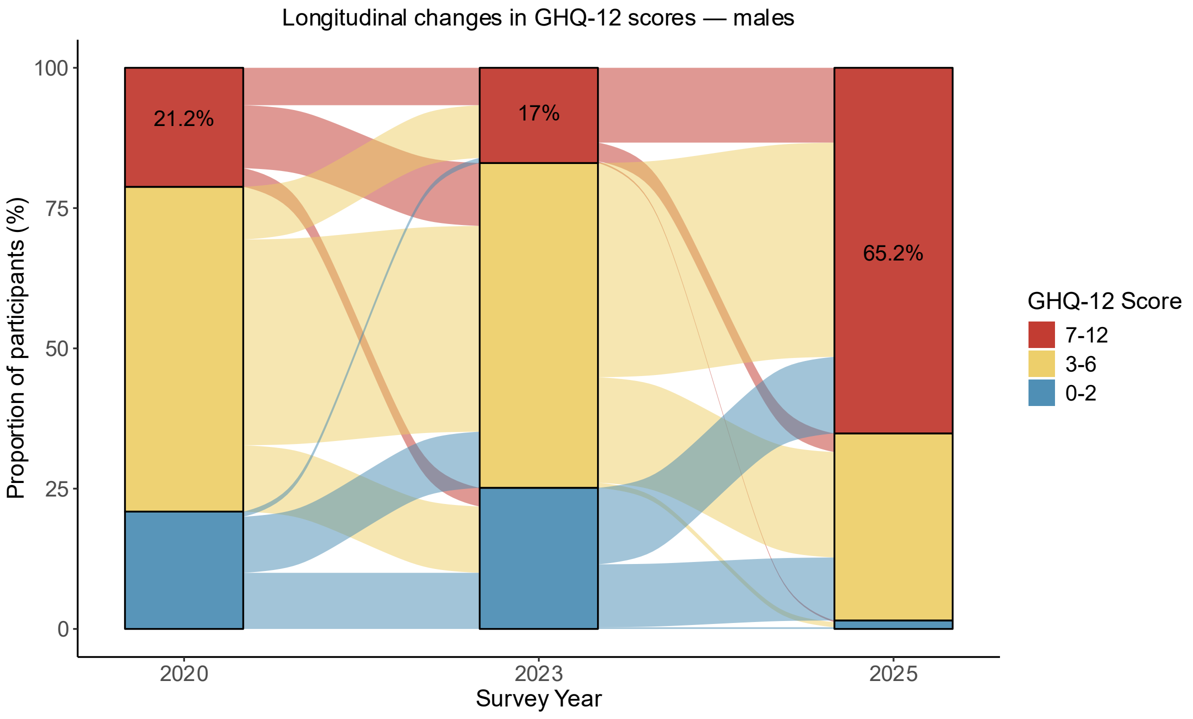

Supplementary Figure 5: Change of General Health Questionnaire (GHQ-12) scores from baseline (2020) through follow-ups in 2023 and 2025 among 330 male participants from Gaza, North Gaza, and Rafah.

GHQ-12 Score Percentiles across Survey Years

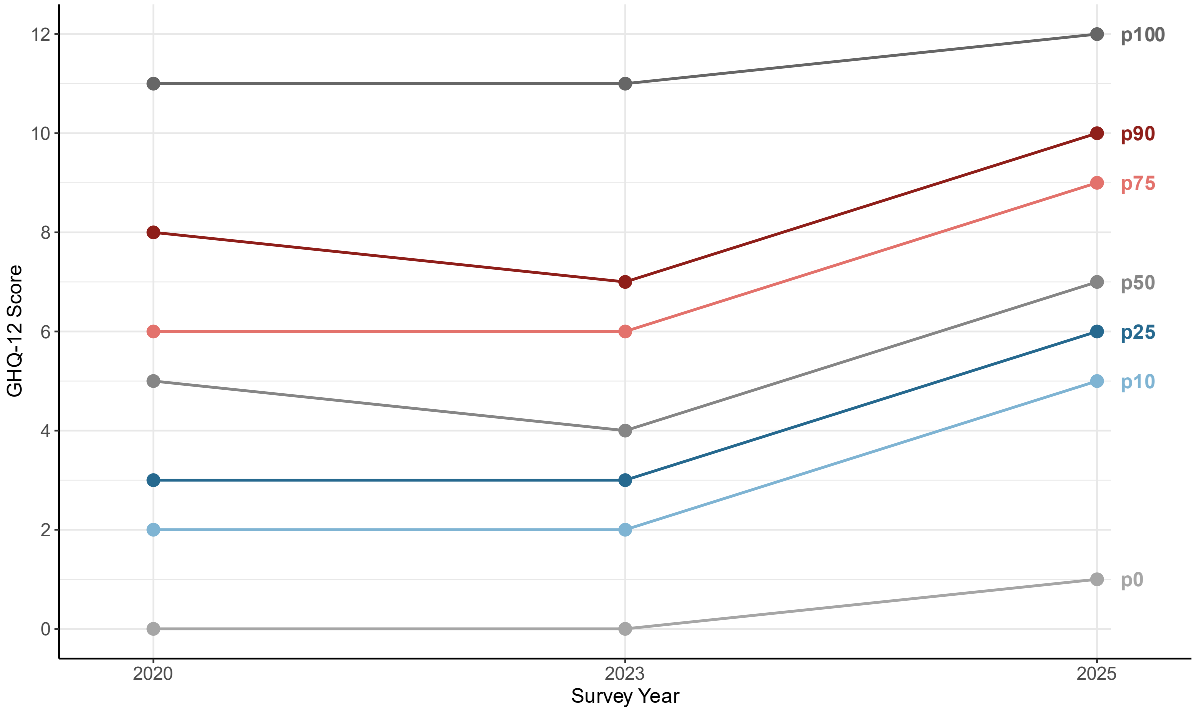

Supplementary Figure 6: Percentiles of the General Health Questionnaire (GHQ-12) score among participants from Gaza, North Gaza and Rafah present in all three surveys (n = 677). Lines indicate selected percentiles, with p0 and p100 representing the minimum and maximum scores, p50 the median, and p10, p25, p75, and p90 the 10th, 25th, 75th, and 90th percentiles, respectively.

Unadjusted regression models

Supplementary Table 4: Unadjusted odds ratios (ORs) with 95% confidence intervals (CIs) from separate mixed-effects logistic regression models, each examining the association between high psychological distress (General Health Questionnaire, GHQ-12 >6) and a single predictor variable. All models account for clustering at both the individual and cluster levels.

Variables	OR	Lower 95% CI	Upper 95% CI
**Survey year**			
2023	0·86	0·64	1·15
2025	12·44	8·99	17·20
**Governorate**			
North Gaza	1·42	1·09	1·84
Rafah	1·66	1·23	2·23
**Gender**			
Male	0·98	0·81	1·18
**Age group**			
60-89	0·76	0·62	0·94
**Civil status**			
Single	1·01	0·66	1·54
**Refugee status**			
Refugee	1·06	0·84	1·34
**Worked in the past 30 days**			
No	1·19	0·93	1·51
**Education**			
Primary	0·95	0·71	1·28
Secondary and higher	0·68	0·50	0·93

Sensitivity analysis using inverse probability weighting

We re-ran the main analysis using inverse probability weighting to minimise the potential impact of attrition bias. First, we estimated the probability of completing the survey using the baseline variables gender, age group, governorate, and refugee status. We then estimated the odds ratios using the same mixed-effects logistic regression model, incorporating stabilised inverse probability weights. The results are shown in the forest plot below.

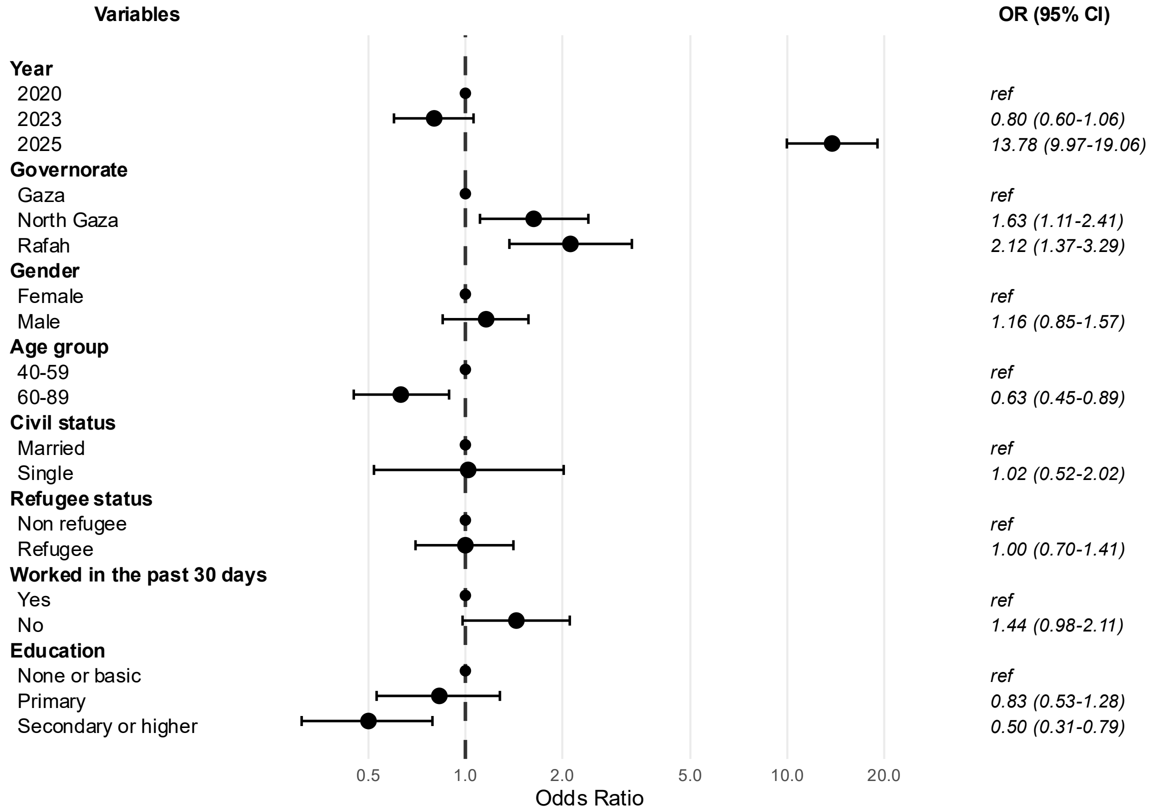

Supplementary Figure 7: Forest plot showing mutually adjusted odds ratios (ORs) for the association between survey year and baseline socio-demographic characteristics with a high level of psychological distress (General Health Questionnaire, GHQ-12 >6), estimated using a multilevel logistic regression model with stabilised inverse probability weights to account for potential attrition bias (n=677). Circles represent ORs and horizontal lines indicate 95% confidence intervals (CIs). Reference categories are indicated as "ref" in the figure.

Answers to GHQ-12 items for the full sample in each survey

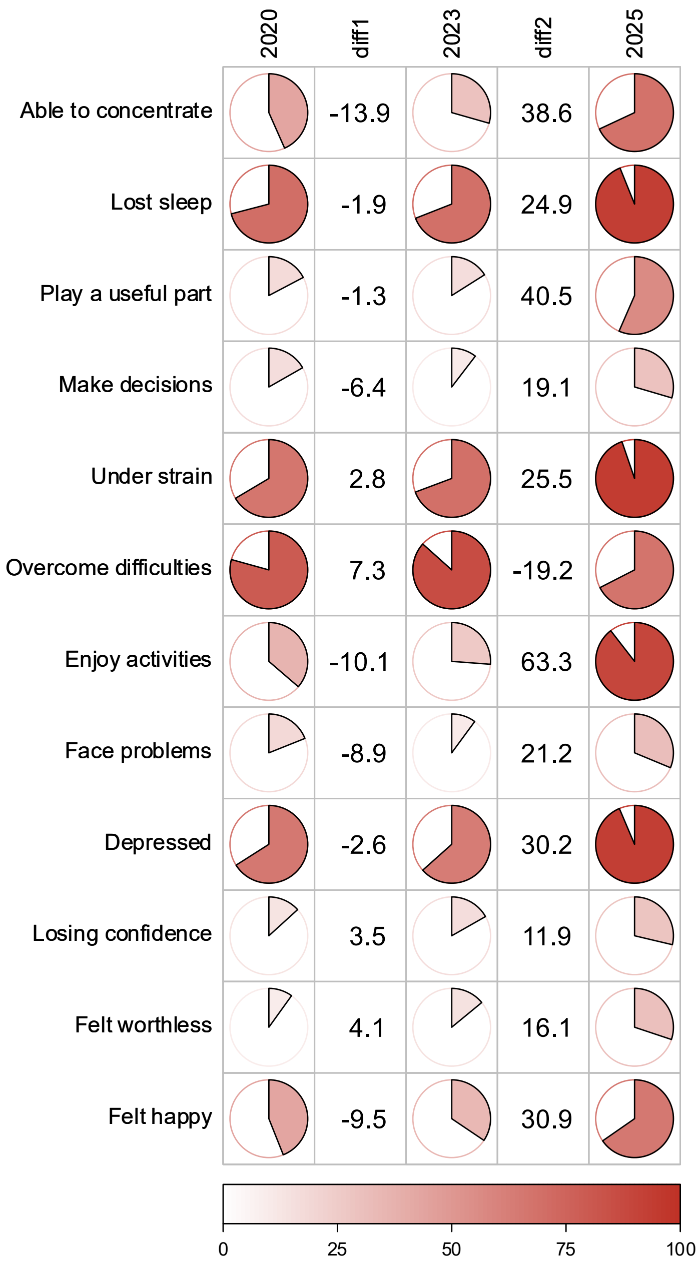

Supplementary Figure 8: Proportion and change in negative responses to individual General Health Questionnaire (GHQ-12) items across survey years among the full available samples from Gaza, North Gaza, and Rafah: 2020 (n=2980), 2023 (n=1615), 2025 (n=704). Darker shades of red represent higher percentages of negative responses, indicating greater psychosocial distress. Changes between surveys are presented as diff1 (percentage point change from 2020 to 2023) and diff2 (percentage point change from 2023 to 2025). Larger and more positive diff values signify greater increases in distress-related responses.
