# Supplementary material for: Changes in psychological distress during conflict escalation in an adult population-based cohort in the Gaza Strip (2020–2025): a longitudinal analysis
Source: eClinicalMedicine. 2025 Nov 24;90:103647. doi: 10.1016/j.eclinm.2025.103647 (PMC12766424; doi:10.1016/j.eclinm.2025.103647)
Supplement: Translated Abstract [file mmc2.pdf]

The following translations in Arabic were submitted by the authors and we reproduce them as supplied. They have not been peer reviewed. Our editorial processes have only been applied to the original abstract in English, which should serve as reference for this manuscript.

Changes in psychological distress during conflict escalation in an adult population-based cohort in the Gaza Strip (2020–2025): a longitudinal analysis

التغيرات في الكرب النفسي أثناء تصعيد الصراع- دراسة على عينة مجتمعيه من البالغين في قطاع غزة (2020-2025): تحليل طولي

الملخص

الخلفية

شهد قطاع غزة صراعاً وحصاراً مطولاً، مما أثقل كاهل البنية التحتية والخدمات الأساسية ومقومات الحياة اليومية. تشكل هذه الضغوط المزمنة تهديداً جسيماً للصحة النفسية. أدى تصعيد الحرب في أكتوبر 2023 إلى نزوح جماعي ودمار واسعي النطاق وخسائر فادحة في الأرواح. بينما تشير الدراسات المقطعية التي أُجريت قبل الحرب إلى معدلات مرتفعة من الكرب النفسي، لا تزال البيانات الطولية التي توثق المسار الزمني للصحة النفسية خلال تصاعد النزاع شحيحة في قطاع غزة وغيره من المناطق المتضررة من الحروب.

المنهجية

تستند هذه الدراسة إلى بيانات طولية من 677 بالغاً تبلغ أعمارهم 40 عاماً فأكثر، شاركوا في ثلاثة مسوح أسرية قائمة على الإبلاغ/ التقرير الذاتي (2020، 2023، 2025) في قطاع غزة. نُفذت المسوح الثلاثة في محافظات غزة وشمال غزة ورفح، وجُمعت الاستجابات في الفترة من 18 مارس إلى 15 يوليو 2020؛ ومن 24 يناير إلى 7 مارس 2023؛ ومن 12 إلى 28 يناير 2025، على التوالي. تم تقييم المتغير الأساسي، وهو الحالة النفسية، في جميع جولات المسح الثلاث باستخدام استبيان الصحة العامة المكون من 12 بنداً (GHQ-12) .سُجلت البنود بطريقة ثنائية (1/0)، بحيث تتراوح الدرجة الإجمالية لـ GHQ-12 من 0 إلى 12، إذ تشير الدرجات الأعلى إلى مستوى أكبر من الكرب النفسي. اعتمدنا عتبة محافظة قدرها GHQ-12>6 لتعريف الكرب النفسي المرتفع، بما يتسق مع الدراسات السابقة في قطاع غزة. وثّقت التحليلات الوصفية التغيرات الزمنية في درجات وبنود GHQ-12. كما قدّر نموذج الانحدار اللوجستي متعدد المستويات العلاقة بين سنة المسح والكرب النفسي المرتفع، مع التحكم في المتغيرات الاجتماعية والديموغرافية.

النتائج

شارك ما مجموعه 677 مشاركاً في جميع المسوح الثلاثة (من أصل 2980 في عام 2020 و1547 في عام 2023). في عام 2020، كان 49٪ و31٪ و20٪ من المشاركين الـ 677 المتبقين يقيمون في محافظات غزة وشمال غزة ورفح، على التوالي. شكّلت النساء نسبة 51٪ (ن=347) من المشاركين، وكانت أعمار 70٪ منهم تتراوح بين 40-59 عاماً. ارتفعت نسبة الكرب النفسي المرتفع من 19.5٪ في عام 2020 و17.4٪ في عام 2023 إلى 67.2٪ في عام 2025. أظهرت النماذج المعدّلة أن احتمالية الكرب النفسي في عام 2025 كانت أعلى بمقدار 12 مرة مقارنة بعام 2020 (نسبة الأرجحية=12.45؛ فاصل/مدى الثقة 95٪: 9.01-17.20). لم تتباين هذه الزيادة عبر الفئات الاجتماعية والديموغرافية الفرعية، وإن كان كبار السن والحاصلون على تعليم ثانوي أو أعلى أقل عرضة لإظهار مستويات مرتفعة من الكرب النفسي.

التفسير

نشأ التضاعف الثلاثي الأخير في معدلات الكرب النفسي الحاد من خلفية اتسمت بوجود عبء صحة نفسية مرتفع للغاية قبل اندلاع الحرب. تؤكد هذه النتائج على الحاجة الملحة لتطبيق خدمات نفسية اجتماعية وصحة نفسية طويلة الأمد، بما في ذلك تعزيز الصلادة، للوقاية من التبعات طويلة المدى على الأجيال الحالية والمقبلة في غزة وغيرها من التجمعات السكانية المتأثرة بالنزاعات. ينبغي للبحوث المستقبلية أن تدرس المسارات طويلة الأجل للصحة النفسية والصلادة، بما في ذلك التأثيرات عبر الأجيال.

التمويل

وزارة التنمية الدولية في المملكة المتحدة، مجلس البحوث الطبية، مجلس البحوث الاقتصادية والاجتماعية، مؤسسة ويلكوم، برنامج صلادة غزة التابع للجنة الدولية للصليب الأحمر، قسم علم الأوبئة والصحة العامة بالمعهد السويسري للصحة الاستوائية والعامة.

الكلمات المفتاحية

الحرب، الصحة النفسية، الكرب النفسي، المجموعة، غزة
